# Books and Authors

**Published:** 1909-01

**Authors:** 


### BOOKS AND AUTHORS.

---

**Diseases and Surgery of the Genitourinary System.** By **Francis S. Watson,** M.D., Senior Visiting Surgeon to the Boston City Hospital, Lecturer on Genitourinary Surgery in the Harvard Medical School, Boston; and **John H. Cunningham, Jr.,** M.D., Assistant Visiting Surgeon to the Boston City Hospital. In two octavo volumes, containing 1,101 pages, with 454 engravings and 47 full-page colored plates, mostly from original drawings. Lea & Febiger, Publishers, Philadelphia and New York, 1908. (Price for the complete work: Extra cloth, $12.00, net; half persian morocco, gilt tops, de luxe, $17.00, net.)

Not until very recently has the study and practice of genitourinary diseases become established as a separate and distinct specialty. Particularly may this be affirmed regarding the surgical treatment of these diseases. This work in its two splendid volumes will be welcomed by the genitourinary specialist because, for the first time, is his specialty dealt with in type so comprehensively, so completely and, withal, so scientifically. But it serves notice on him that he can no longer be classed as a specialist unless he is prepared to do all the surgery of the genitourinary tract, from incising the meatus to extirpation of the kidney, from catheterisation to prostatectomy. In other words, the genitourinary specialist of today must, perforce, equip himself to meet every phase of the work, from the simplest therapeutic to the gravest surgical procedure. This is the work that teaches every detail of the topic, every minor point, as well as every major principle. It is valuable also to the general practitioner, especially he who must do everything in medicine and surgery that comes to him.

The anatomy of the genitourinary tract and its environment, is developed step by step, as the several regions are approached and as each operation is described. The authors, in addition to their own studies in the anatomy of the parts, have drawn upon Sobotta, Deaver, Testut, and others,—all men of great distinction in anatomical study and as teachers. Watson pays great compliment to Deaver, which is eminently deserved. Especially is this true with reference to Deaver's excellent work in operations on the prostate.

If the anatomic studies and delineations merit praise, what shall be said of the operative procedures and depictions themselves? Nowhere in the literature has anything appeared so complete, so clear, so understandable even to the novice, as in this treatise, not only as relates to its descriptive text, but in its drawings and illustrations in general. While it must be conceded that the treatise is very strong in all the major operations on the genitourinary tract, we have been impressed with the further fact that it deals masterfully with those operations which we are accustomed to call minor, such for example, as the surgery of the prepuce, plastic operations on the penis, internal urethrotomy and divulsion for the relief of stricture, and the like.

In describing the technic of many of these minor details, Watson is superb. He tells of the instruments needed and how to use them. We may cite, as an illustration, his description of passing the sound, which is so complete that even the tyro should not fail; and he supports the descriptive text with such perfection of pictures that one can almost imagine he is in the author's clinic, witnessing the demonstration on a real patient.

The first volume considers the external genitals, the prostate, and the bladder, contains 627 imperial octavo pages with 339 illustrations in the text, besides several plates. The second volume deals with the kidneys and ureters, contains 474 pages, with 115 illustrations in the text, and also many plates in color and half-tone, all constituting a treatise rich in scientific material and filled with the perfection of pictorial art.

Watson, the author-in-chief, has been engaged about seven years in the preparation of this work. After being occupied four years diligently, he foresaw that he would not be able to finish it without aid within the specified time, whereupon he called to his assistance John H. Cunningham, Jr., his former house surgeon, and these two together, master and pupil, have wrought out and presented to the profession an enduring contribution to the literature of medicine. The illustrations, profuse though they are, present a striking object lesson. They are realistic exhibitions of the conditions portrayed, many being original and all recent. The authors are fortunate in having such talented artists at command. The entire treatise is a monument to the skill and talent of American authors, artists, and publishers.

---

**The Medical Record Visiting List or Physicians' Diary for the Year 1909.** New and revised edition New York: William Wood & Company, Medical Publishers, 51 Fifth Avenue.

This book has been before the profession so long and in the hands of such a multitude of physicians each year, that nearly everybody interested is familiar with it. For the information of those unacquainted with its make-up, we invite attention to its table of contents:

Calendar; Estimation of the probable duration of pregnancy; Approximate equivalents of temperature, weight, capacity, measure, etc.; Maximum adult doses by the mouth, in apothecaries' and decimal measures; Drops in a fluid drachm; Solutions for subcutaneous injection; Solutions in water for atomisation and inhalation; Miscellaneous facts; Emergencies; Surgical antisepsis; Disinfection; Dentition; Table of signs; Visiting list with special memoranda; Consultation practice; Obstetric engagements; Record of obstetrical practice; Record of vaccination; Register of deaths; Nurses' addresses; Addresses of patients and others; Cash account.

The prices of regular lists are: for 60 patients a week, with or without dates, handsomely selected red or black, morocco binding, $1.50; for 30 patients a week, with or without dates, same style, $1.25; also a few special 90 patient lists, $2.00.

These visiting lists are fitted into genuine seal and calf skin wallets, making the most elegant books of the kind ever offered to the profession. The lists proper are in two books of six months each, and are removable from the wallets. They are thus much less bulky for the pocket. They are also economical inasmuch as the leather covers may be made to do service for several years. We unhesitatingly recommend the Medical Record visiting list as one of the most useful, as well as among the handsomest offered in the market.

---

**Diseases of Infancy and Childhood. By Louis Fischer,** M.D., Attending Physician to the Willard Parker and Riverside Hospitals, New York. Second edition. Octavo, pp, 979. With 403 illustrations, several in colors, and 27 full page half-tone and color plates. Philadelphia: F. A. Davis Co. Cloth, $6.50.

A remarkable fact relating to this treatise is that a second edition was called for within six months,—a sure indication of its value. The author is to be congratulated on such signal success. Notwithstanding the sudden demand for a new edition, careful revision has been made even to the extent of changes in many of the articles. Others have been elaborated, as in the treatment of nephritis the salt free diet has been added. Tuberculin innoculation as an aid to diagnosis has been added, and other changes have been made, including new and improved illustrations, bringing it abreast of the immediate present in all essential particulars. It is one of the best textbooks of its kind.

